# Diffuse Gonococcal Infection (DGI) in a Patient with Treatment-Refractory Acetylcholine Receptor Antibody-Positive (AChR+) Generalized Myasthenia Gravis (gMG) Treated with Eculizumab

**DOI:** 10.1155/2021/9713413

**Published:** 2021-06-14

**Authors:** Nakul Katyal, Latika Nirola, Naureen Narula, Raghav Govindarajan

**Affiliations:** ^1^Department of Neurology, University of Missouri, Columbia, MO, USA; ^2^Guru Gobind Singh Medical College and Hospital, Faridkot, Punjab, India; ^3^Department of Pulmonology and Critical Care Medicine, Staten Island University Hospital, New York, NY, USA

## Abstract

Patients receiving complement inhibitor, eculizumab, are at high risk for infections with encapsulated organisms such as *Neisseria* due to impaired opsonophagocytic activity. Impaired complement immunity may increase the risk for dissemination of asymptomatic *Neisseria gonorrhoeae*. Disseminated Gonococcal Infection (DGI) is a rare but potentially life-threatening complication associated with eculizumab. Physicians should obtain adequate sexual histories from the patients and educate them on safe sexual practices. Here, we describe a case of DGI in a 32-year-old African American female patient with acetylcholine receptor antibody-positive (AChR+) generalized myasthenia gravis (gMG), receiving eculizumab.

## 1. Introduction

Eculizumab is a recombinant humanized monoclonal antibody that binds to complement component C5 and prevents conversion to the proinflammatory C5a and eventually membrane attack complex (MAC) formation [1]. Eculizumab has been approved for management of patients with paroxysmal nocturnal hemoglobinuria (PNH) and atypical haemolytic uremic syndrome (HUS) [2]. In a phase 3, randomized, double-blind, placebo-controlled study (REGAIN) and its open-label extension, eculizumab was shown to be effective in patients with treatment-refractory acetylcholine receptor antibody-positive (AChR+) generalized myasthenia gravis (gMG) [3]. In 2017, eculizumab was approved by the Food and Drug Administration (FDA) for the treatment of AChR+ gMG [4]. Patients receiving eculizumab are at high risk for infections with encapsulated organisms such as *Neisseria* due to impaired opsonophagocytic activity [5]. Administration of meningococcal vaccines, therefore, is recommended before beginning treatment [6]. Despite vaccination, meningococcal disease has been reported in recipients of eculizumab [7, 8]. In vitro data have shown that eculizumab impairs meningococcal killing in whole blood even in subjects vaccinated against the relevant meningococcal serogroup [5, 7, 8]. Similarly, impaired complement immunity with eculizumab may increase the risk for dissemination of *Neisseria gonorrhoeae*. Here, we describe a case of Disseminated Gonococcal Infection (DGI) in a 32-year-old African American female patient with AChR+ gMG receiving eculizumab.

## 2. Case Report

A 32-year-old African American female with a past medical history of deep vein thrombosis (DVT) and AChR+ gMG status after thymectomy, on pyridostigmine (60 mg three times a day) and eculizumab, presented to the Emergency Room (ER), a week after sustaining a mechanical fall while walking carrying her child on an ice-covered pavement. During the fall, her child landed on her left knee. The patient was first started on eculizumab 2 years ago (June 2018) after receiving both the monovalent and quadrivalent meningococcal vaccines as per Advisory Committee on Immunization Practices (ACIP) guidelines. She was on eculizumab for a year but then stopped it (June 2019) due to significant improvement in her MG symptoms. She was off all MG treatments for a year and half but was restarted on eculizumab (Nov 2020) due to worsening fatigue, axial weakness, and dysphagia. Her last dose was 8 days before presentation to the ER.

At the time of presentation to the ER, she complained of 8/10, sharp pain, localized to the left knee, worse with movement, and better with rest. Her vital signs were unremarkable. Examination was remarkable for left knee tenderness to palpation and limited active and passive range of motion. Laboratory workup showed elevated White Blood Cell (WBC) count to 12,000/L with 72% neutrophils, elevated Erythrocyte Sedimentation Rate (ESR) to 111 mm/hour, elevated alkaline phosphatase to 162 Units/L, and C reactive protein (CRP) to 17.19 mg/dl. Urinary *N. gonorrhoeae* testing with a Nucleic Acid Amplification Technique (NAAT) showed a positive result. She denied any change in her sexual contacts or history of Sexually Transmitted Diseases (STDs). We suspect she acquired the infection from her husband who was later tested positive. X-ray of the left knee showed moderate effusion without evidence of fracture. Arthrocentesis of the left knee showed a cloudy, yellow-colored fluid with a WBC of 50,000/mcL, with 95% neutrophils, Red Blood Cell (RBC) count of 3000/mcL, and no crystals. She was diagnosed with septic arthritis of the left knee and underwent irrigation and debridement of the left knee that drained 5 cc of purulent fluid. Postoperatively, the patient was started on vancomycin (1250 mg Intravenous (IV) every 8 hours) and cefepime (2 gram IV every 8 hours). The left knee aspirate and left knee operative cultures remained negative.

On day 2 of hospitalization, blood culture from day 0 grew Gram-Positive Cocci (GPC) in the cluster, later confirmed as *N. gonorrhoeae*. She was then diagnosed with Disseminated Gonococcal Infection (DGI) likely related to recent eculizumab use (8 days before presentation). After obtaining antibiotic sensitivity testing results, on day 3 of hospitalization, vancomycin and cefepime were deescalated to ceftriaxone. She developed facial swelling after receiving the first dose of ceftriaxone that was treated with IV diphenhydramine. The patient was then switched to ertapenem 1 gram IV every 24 hours which she tolerated without any side effects. Further testing for STDs including Human Immunodeficiency Virus (HIV), syphilis, trichomonas, and chlamydia was negative. A repeat blood culture from day 2 was negative, and the patient was discharged home to complete four weeks of ertapenem therapy which she has since completed. The repeat ESR after 4 weeks trended down to 62.

## 3. Discussion

Our study described the complication of DGI in a patient with gMG on eculizumab. Previous studies have described similar complications of DGI in patients with PNH and HUS on eculizumab [9]. To our best knowledge, our case is the first one to describe this complication in a patient with AChR+ gMG, treated with eculizumab.

Gonorrhea is the second most commonly reported notifiable disease in the United States [9, 10]. In 2018, a total of 583,405 cases of gonorrhea were reported in the United States [10]. Persons aged 15–44 years accounted for 91.6% of the reported gonorrhea cases [10]. Although urethral gonorrhea can cause profuse discharge and pain, gonococcal infections of the cervix, pharynx, and rectum are often asymptomatic [9–11]. In approximately 0.5–3% of gonorrhea cases, the bacterium can enter the bloodstream and cause DGI [9, 12]. Patients receiving eculizumab are at high risk for infections with encapsulated organisms such as *N. gonorrhoeae* due to impaired opsonophagocytic activity [5].

A case study by Crew et al. reviewed the FDA pre- and postmarketing safety reports of gonococcal infections among patients receiving eculizumab [9]. The study described 9 cases of gonococcal infections in patients receiving eculizumab for either PNH or HUS [9]. Eight out of nine patients were females, and seven out of nine were <30 years of age [9]. Eight of the nine patients were classified as having DGI and were hospitalized [9]. A diagnostic workup of cerebrospinal fluid sampling was conducted on three patients for meningitis, but none was diagnosed, one patient had a trans-thoracic echocardiogram which was negative for valvular vegetation, and two patients required vasopressor support for the concern of septic shock [9]. Seven patients continued the therapy after the infection while one discontinued [9]. All gonococcal infections resolved except in one case where the patient had developed endocarditis and thrombotic complications and died [9].

Vaccines containing group B *N. meningitidis* outer-membrane vesicles (OMV) may provide protection against a significant proportion of *N. gonorrhoeae*, but the mechanisms for the same have not yet been elucidated and the vaccination has not been approved for use in gonococcal infection [13]. Therefore, despite vaccination against *N. meningitidis*, these patients are still at a potential risk for developing gonococcal disease. CDC recommends annual screening for gonorrhea in all young sexually active women <25 years of age and older women who are at an increased risk [9, 11]. As of now, the screening recommendations for gonorrhea in patients receiving eculizumab do not differ from those for the general population [9]. In our case, the patient acquired the infection from her significant other who was later educated and treated of *N. gonorrhoeae*. Physicians are encouraged to obtain adequate sexual histories of patients and educate them on safe sexual practices, regular use of condoms, and avoiding unprotected sexual contact [9]. In addition, patients should be educated about the increased risk of gonococcal infection and DGI with eculizumab. Healthcare professionals should keep DGI in their differential diagnosis, especially in a patient with skin or joint involvement [9].

## 4. Conclusions

DGI is a rare but potentially life-threatening complication associated with use of complement inhibitor, eculizumab. Physicians should obtain adequate sexual histories from the patients and educate the patients and, if possible, their partners on safe sexual practices.

## Data Availability

The data used to support the findings of this study are included within the article.

## Abbreviations

AChR+: Acetylcholine receptor antibody-positive
gMG: Generalized myasthenia gravis
DGI: Disseminated gonococcal infection
PNH: Paroxysmal nocturnal hemoglobinuria
HUS: Atypical haemolytic uremic syndrome
MAC: Membrane attack complex
FDA: Food and Drug Administration
DVT: Deep vein thrombosis
ER: Emergency room
STD: Sexually transmitted disease
CRP: C-reactive protein
ESR: Erythrocyte sedimentation rate
NAAT: Nucleic acid amplification technique
WBC: White blood cell
RBC: Red blood cell.
